# Abdominal Binding Improves Neuromuscular Efficiency of the Human Diaphragm during Exercise

**DOI:** 10.3389/fphys.2017.00345

**Published:** 2017-05-31

**Authors:** Sara J. Abdallah, David S. Chan, Robin Glicksman, Cassandra T. Mendonca, Yuanming Luo, Jean Bourbeau, Benjamin M. Smith, Dennis Jensen

**Affiliations:** ^1^Clinical Exercise and Respiratory Physiology Laboratory, Department of Kinesiology and Physical Education, McGill UniversityMontréal, QC, Canada; ^2^State Key Laboratory of Respiratory Disease, Guangzhou Medical UniversityGuangzhou, China; ^3^Department of Medicine, Respiratory Division, McGill UniversityMontréal, QC, Canada; ^4^Respiratory Epidemiology and Clinical Research Unit, Montréal Chest Institute, McGill University Health CentreMontréal, QC, Canada; ^5^Meakins-Christie Laboratories, Research Institute of the McGill University Health CentreMontréal, QC, Canada; ^6^McConnell Centre for Innovative Medicine, Research Institute of the McGill University Health CentreMontréal, QC, Canada; ^7^Centre for Outcomes Research and Evaluation, Research Institute of the McGill University Health CentreMontréal, QC, Canada; ^8^Translational Research in Respiratory Diseases Program, Research Institute of the McGill University Health CentreMontréal, QC, Canada; ^9^Research Centre for Physical Activity and Health, McGill UniversityMontréal, QC, Canada

**Keywords:** breathlessness, exercise, abdominal binding, neuromuscular efficiency, diaphragm

## Abstract

We tested the hypothesis that elastic binding of the abdomen (AB) would enhance neuromuscular efficiency of the human diaphragm during exercise. Twelve healthy non-obese men aged 24.8 ± 1.7 years (mean ± *SE*) completed a symptom-limited constant-load cycle endurance exercise test at 85% of their peak incremental power output with diaphragmatic electromyography (EMGdi) and respiratory pressure measurements under two randomly assigned conditions: unbound control (CTRL) and AB sufficient to increase end-expiratory gastric pressure (Pga,ee) by 5–8 cmH_2_O at rest. By design, AB increased Pga,ee by 6.6 ± 0.6 cmH_2_O at rest. Compared to CTRL, AB significantly increased the transdiaphragmatic pressure swing-to-EMGdi ratio by 85–95% during exercise, reflecting enhanced neuromuscular efficiency of the diaphragm. By contrast, AB had no effect on spirometric parameters at rest, exercise endurance time or an effect on cardiac, metabolic, ventilatory, breathing pattern, dynamic operating lung volume, and perceptual responses during exercise. In conclusion, AB was associated with isolated and acute improvements in neuromuscular efficiency of the diaphragm during exercise in healthy men. The implications of our results are that AB may be an effective means of enhancing neuromuscular efficiency of the diaphragm in clinical populations with diaphragmatic weakness/dysfunction.

## Introduction

Diaphragm muscle weakness/dysfunction is pervasive in many clinical populations, including chronic obstructive pulmonary disease (COPD), interstitial lung disease, heart failure, neuromuscular disease, critical illness and mechanical ventilation, and spinal cord injury (SCI; Baydur, 1991; Nishimura et al., 1994; Tantucci et al., 1994; Polkey et al., 1996; Baydur et al., 2001; Meyer et al., 2001; Laghi and Tobin, 2003; Brown et al., 2006; Kabitz et al., 2006, 2007; Petrof et al., 2010; West et al., 2012b). In these patient populations, diaphragm muscle weakness/dysfunction has been linked to increased breathlessness, impaired exercise tolerance, prolonged and difficult weaning from mechanical ventilation, and adverse health outcomes, including quality of life and death (Laghi and Tobin, 2003). It follows that non-disease specific interventions capable of increasing the pressure generating capacity of the diaphragm may have important clinical and pathophysiological implications. With the exception of inspiratory muscle training (Budweiser et al., 2006; Geddes et al., 2008; Moodie et al., 2011; Berlowitz and Tamplin, 2013; Smart et al., 2013; Martin-Valero et al., 2014) and the Ca^2+^ sensitizing agent, Levosimendan (van Hees et al., 2009; Doorduin et al., 2012), few generalized interventions exist to improve the force generating capacity of the human diaphragm.

Accumulating evidence from studies in health (Koulouris et al., 1989; West et al., 2012a) and SCI (Goldman et al., 1986; Hart et al., 2005; West et al., 2012a) suggest that elastic binding of the abdomen (AB) significantly increases maximal voluntary (e.g., sniff) and involuntary (e.g., twitch) pressure generating capacity of the diaphragm, presumably by reducing abdominal wall compliance, improving the operating length of the diaphragm due to its ascent to a more mechanically advantageous (cephalad) end-expiratory position, increasing intra-abdominal pressure, increasing the area of diaphragmatic apposition to the rib cage and/or increasing diaphragm-rib cage insertional forces (McCool et al., 1986; Koo et al., 2015). A series of studies by West et al. (2012a, 2014a,b) recently reported that AB sufficient to increase end-expiratory gastric pressure (Pga,ee) by an average of ~8 cmH_2_O at rest in athletes with cervical SCI increased transdiaphragmatic twitch pressures by ~40% relative to the unbound control condition. In those studies, AB-induced improvements in diaphragmatic function were associated with concurrent improvements in static lung volumes and capacities; cardiac output at rest; the behavior of dynamic operating lung volumes during exercise; and selected measures of field-based exercise performance.

To our knowledge, only two studies have examined the impact of AB on exercise physiological responses in healthy adults (Vanmeenen et al., 1984; Hussain et al., 1985). Vanmeenen et al. (1984) examined the effects of decreasing vital capacity by ~30% through the application of an inelastic canvas corset around the abdomen (extending from the xyphoid process to the hips, thus encompassing the lower five ribs) on exercise physiological responses in 11 healthy men. In that study, AB impaired ventilatory and cardiovascular responses to exercise with attendant reductions in exercise performance, consistent with the established effects of external thoracic restriction on exercise physiological responses in healthy men (Harty et al., 1999; O'Donnell et al., 2000; Miller et al., 2002; Mendonca et al., 2014). A similar study by Hussain et al. (1985) found that applying an inelastic corset around the abdomen of five healthy men as tightly as possible while interfering minimally with ribcage movements, caused a “mild” restrictive lung deficit; significantly increased transdiaphragmatic pressure (Pdi) swings during exercise; and had no effect on exercise tolerance or an effect on ventilation ($\dot{\text{V}}$_E_), breathing pattern and diaphragmatic electromyography (EMGdi) responses to exercise. While the study by Hussain et al. (1985) suggested that AB has the potential to enhance neuromuscular efficiency of the human diaphragm during exercise (i.e., increase ratio of Pdi-to-EMGdi), the authors did not (1) control for the degree of abdominal compression applied; (2) account for the possibility that the “mild” restrictive lung deficit imposed by AB may have offset the potential benefits of enhanced neuromuscular efficiency of the diaphragm on exercise tolerance; and/or (3) examine the simultaneous effect of AB on cardiac, metabolic, dynamic operating lung volume, and breathlessness responses to exercise.

The purpose of this study was to examine the effect of AB sufficient to increase Pga,ee by 5–8 cmH_2_O at rest on cardiac, metabolic, ventilatory, breathing pattern, dynamic operating lung volume, EMGdi, respiratory pressure, and breathlessness responses during high-intensity constant-load cycle endurance exercise testing in healthy men.

## Materials and methods

### Study design

This was a single-center, controlled, randomized, crossover study wherein eligible men participated in three testing visits over a period ≤2 weeks. Visit 1 included screening of medical history, spirometry, and a symptom-limited incremental cycle exercise test to determine peak power output (PPO). Visits 2 and 3 included spirometry and a symptom-limited constant-load cycle endurance exercise test at 85% of PPO with added measurements of EMGdi and respiratory pressures under two randomly assigned conditions: unbound control (CTRL) and AB. Although the conditions could not be blinded to the participants and investigators, the participants were naïve to the expected outcomes of the study. Visit 1–3 were separated by ≥24 h and conducted at the same time of day (±1 h) for each participant. Participants were instructed to avoid alcohol, caffeine, heavy meals, and strenuous exercise on each test day. The study was approved by the Institutional Review Board of the Faculty of Medicine at McGill University (A04-M42-12B) in accordance with the *Declaration of Helsinski*. Written informed consent was obtained from all participants prior to study initiation.

### Participants

Participants included 12 non-smoking, non-obese men aged 18–40 years with normal spirometry [forced expiratory volume in 1 s (FEV_1_) ≥80% predicted (Tan et al., 2011) and FEV_1_-to-forced vital capacity ratio ≥70%] and no known or suspected cardiovascular, respiratory, metabolic, musculoskeletal, endocrine, and/or neuromuscular disorder(s).

### Abdominal binding

As described in detail elsewhere (West et al., 2012a), a binder made primarily of flexible neoprene (493R Universal Back Support; McDavid Inc., Woodridge, IL, USA) was individually sized and fitted with participants in the upright position and with the binder's upper edge below the costal margin so that it interfered minimally with rib-cage movement. The desired degree of abdominal compression—defined as an increase in Pga,ee of 5–8 cmH_2_O during steady-state breathing while seated on a chair at rest prior to exercise—was achieved by tightening Velcro fasteners at the front of the binder. An earlier study by West et al. (2012a) found that this level of abdominal compression optimized pulmonary function and twitch Pdi responses at rest in healthy adults and among individuals with cervical SCI.

### Spirometry

Spirometry was performed using automated equipment (Vmax Encore™, CareFusion, Yorba Linda, CA, USA) according to recommended techniques (Miller et al., 2005).

### Cardiopulmonary exercise testing

Symptom-limited exercise tests were performed on an electronically braked cycle ergometer (VIAsprint 150P; Ergoline, Bitz, Germany) using a cardiopulmonary exercise testing system (Vmax Encore™, CareFusion). Incremental exercise tests consisted of a steady-state resting period of ≥6 min, followed by 25 W increases in power output (starting at 25 W) every 2 min: PPO was defined as the highest power output that the participant was able to sustain for ≥30 s. Constant-load exercise endurance tests consisted of a steady-state resting period of ≥6 min followed by a step increase in power output to 85% PPO.

Standard cardiopulmonary exercise test parameters were collected breath-by-breath (Mendonca et al., 2014; Schaeffer et al., 2014), while heart rate (HR), stroke volume (SV), and cardiac output (CO) were assessed using an impedance cardiograph (PhysioFlow®; NewMeDx, Bristol, PA, USA) that provides an acceptable and non-invasive evaluation of CO during symptom-limited cycle exercise testing in both health and disease (Charloux et al., 2000; Richard et al., 2001). Inspiratory capacity (IC) maneuvers were performed at rest, within the last 30 s of every 2 min interval during exercise and at end-exercise (Mendonca et al., 2014; Schaeffer et al., 2014). Assuming that total lung capacity does not change during exercise with and without AB in normal males (Stubbing et al., 1980), changes in IC and inspiratory reserve volume [IRV = IC – tidal volume (V_T_)] reflect changes in dynamic end-expiratory and end-inspiratory lung volume, respectively.

Breath-by-breath measures of the root mean square of EMGdi (EMGdi,rms) and of esophageal (Pes), gastric (Pga), and transdiaphragmatic pressure (Pdi = Pga – Pes) were recorded from a gastro-esophageal electrode-balloon catheter (Guangzhou Yinghui Medical Equipment Ltd., Guangzhou, China) and analyzed using published methods (Mendonca et al., 2014; Schaeffer et al., 2014). Maximum voluntary EMGdi,rms was identified as the largest of all EMGdi,rms values obtained from IC maneuvers performed either at rest or during exercise. Tidal swings in Pes (Pes,tidal), Pga (Pga,tidal), and Pdi (Pdi,tidal) were calculated as the difference between peak tidal inspiratory and peak tidal expiratory Pes, Pga, and Pdi, respectively. The ratio of Pdi,tidal-to-EMGdi,rms was used as an index of neuromuscular efficiency of the diaphragm.

Using Borg's 0–10 category ratio scale, participants rated the intensity of their breathing overall and the intensity of their leg discomfort at rest, within the last 30 s of every 2 min interval during exercise and at end-exercise (Borg, 1982). Breathing overall (hereafter referred to as breathlessness) was defined as “the global awareness of your breathing,” which is consistent with the American Thoracic Society's recommendation that the definition of breathlessness should be neutral with respect to any particular quality of breathing (Parshall et al., 2012). Leg discomfort was defined as the “difficulty associated with pedaling.” Participants were asked to verbalize their main reason(s) for stopping exercise; quantify the percentage contribution of breathlessness and leg discomfort to exercise cessation; and identify qualitative phrases that best described their breathlessness at end-exercise (O'Donnell et al., 2000).

### Analysis of exercise end-points

All physiological parameters were averaged in 30 s intervals at rest and during exercise. These parameters, averaged over the first 30 s of every 2 min interval during exercise, were linked with IC and symptom measurements collected during the last 30 s of the same minute. Three main time points were used for the evaluation of measured parameters: (1) *pre-exercise rest*, defined as the average of the last 60 s of the steady-state period after ≥3 min of breathing on the mouthpiece while seated on the cycle ergometer before the start of exercise; (2) *isotime*, defined as the average of the first 30 s of the 2nd min of the highest equivalent 2 min interval of constant-load cycle exercise completed by a given participant with and without AB; and (3) *peak exercise*, defined as the average of the last 30 s of loaded pedaling. Exercise endurance time (EET) was the duration of loaded pedaling.

### Statistical analysis

Two-tailed paired *t*-tests were used to examine the effects of AB vs. CTRL on spirometric parameters, maximal voluntary EMGdi,rms, and the percentage contribution of breathlessness and leg discomfort to exercise cessation. A two-way repeated measures analysis of variance with Tukey's HSD *post-hoc* test was used to examine the effect of AB vs. CTRL on physiological and perceptual parameters measured at rest, at standardized submaximal time points during exercise (including isotime) and at peak exercise. All analyses were performed using SigmaStat®, version 3.5 (Systat® Software, San Jose, CA, USA) and statistical significance was set at *p* < 0.05. Data are presented as means ± SEM.

## Results

### Participants, abdominal binding, and spirometry

Participants were healthy, young (24.8 ± 1.7 years), non-obese (body mass index = 23.1 ± 0.6 kg × m^−2^) and non-smoking men with normal cardiorespiratory fitness: symptom-limited peak rate of O_2_ consumption ($\dot{\text{V}}$O_2_) of 55.1 ± 2.2 ml × kg × min^−1^ or 121 ± 6% predicted (Jones et al., 1985); and PPO of 267 ± 18 W or 109 ± 5% predicted (Jones et al., 1985). By design, AB increased Pga,ee by 6.6 ± 0.6 cmH_2_O above its baseline value during the AB visit, but had no effect on spirometric parameters compared with CTRL (Table 1).

**Table 1:** **Effect of abdominal binding (AB) on spirometric pulmonary function test parameters at rest in healthy men**.

**Parameter**	**Control**	**AB**
FVC, L	5.48 ± 0.22	5.35 ± 0.25
FEV_1_, L (% predicted)	4.41 ± 0.19 (95 ± 3)	4.27 ± 0.21 (92 ± 4)
FEV_1_/FVC, %	81 ± 2	80 ± 2
PEF, L × s^−1^	10.4 ± 0.5	9.8 ± 0.6
FEF_25−75%_, L × s^−1^	4.22 ± 0.33	4.04 ± 0.34

*Values are means ± SEM. FVC, forced vital capacity; FEV_1_, forced expiratory volume in 1-sec; PEF, peak expiratory flow; FEF_25−75%_, forced expiratory flow between 25 and 75% of the FVC maneuver*.

### Physiological and perceptual responses to exercise

The order of experimental conditions was balanced such that 7 of the 12 participants were randomized to exercise with AB at Visit 2. To rule out a potentially confounding order effect on exercise performance, we compared EET between Visits 2 and Visits 3, irrespective of experimental condition and found no statistically significant difference: 9.7 ± 1.0 vs. 9.0 ± 1.2 min, respectively (*p* = 0.290).

**Figure 1:**
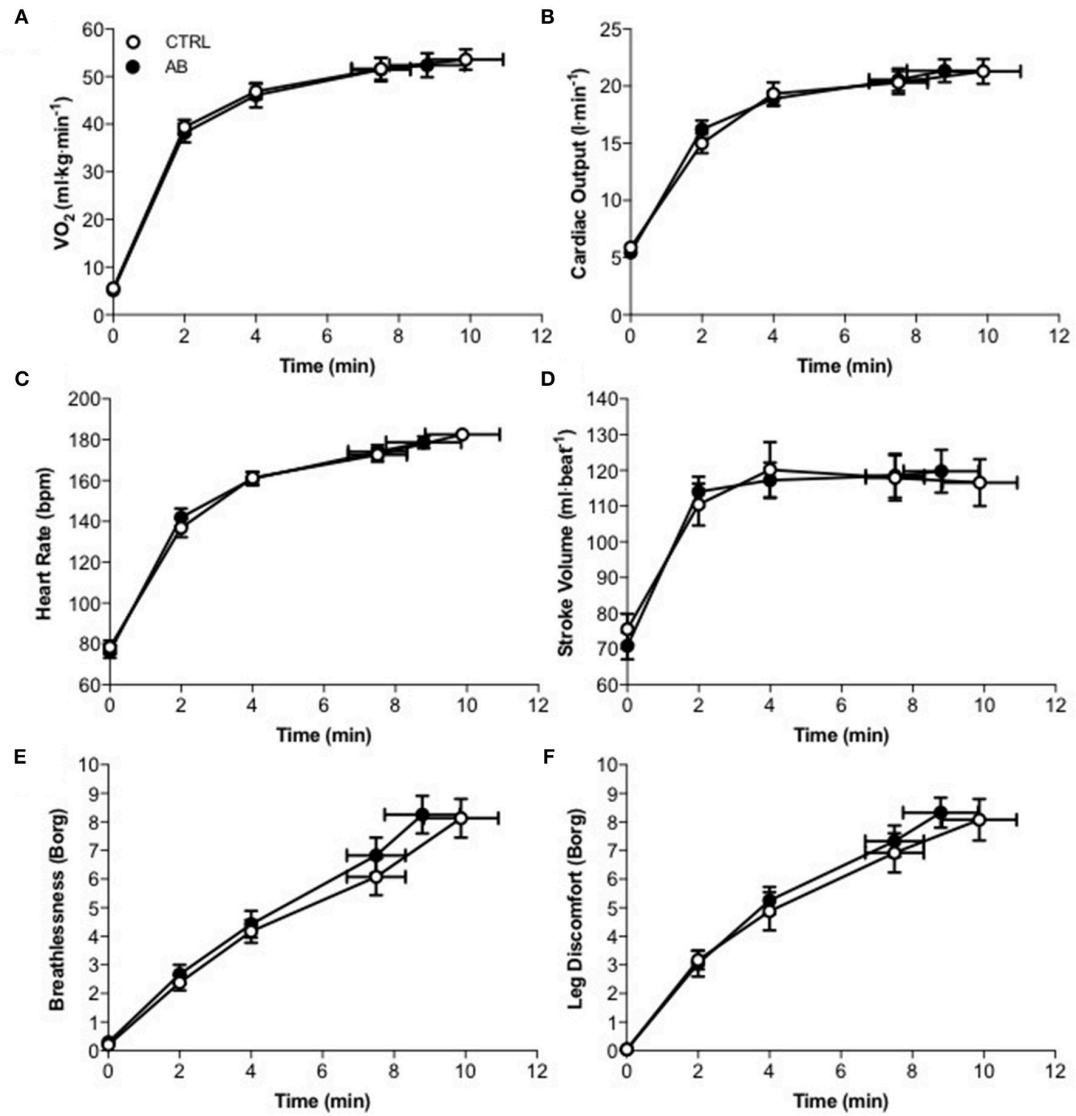
**Effect of abdominal binding (AB) vs. control (CTRL) on (A)** the rate of oxygen consumption ($\dot{\text{V}}$ O_2_), **(B)** cardiac output **(C)** heart rate, **(D)** stroke volume, **(E)** breathlessness, and **(F)** leg discomfort responses during constant-load cycle endurance exercise testing at 85% of peak incremental power output in healthy men. Values are means ± SEM.

**Figure 2:**
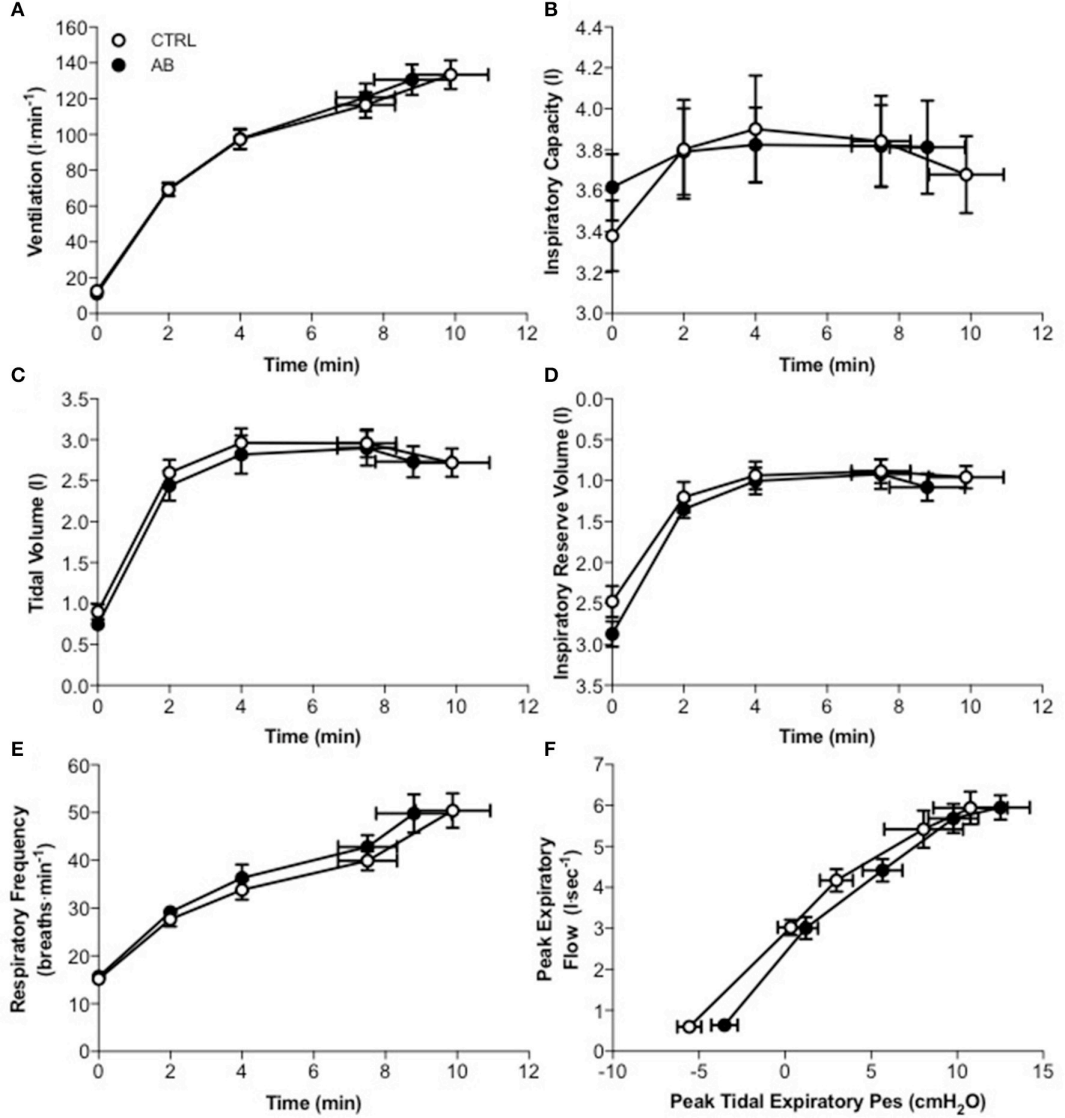
**Effect of abdominal binding (AB) vs. control (CTRL) on (A)** ventilation, **(B)** inspiratory capacity, **(C)** tidal volume, **(D)** inspiratory reserve volume, **(E)** respiratory frequency, and **(F)** peak expiratory flow vs. peak tidal expiratory esophageal pressure (Pes) responses during constant-load cycle endurance exercise testing at 85% of peak incremental power output in healthy men. Values are means ± SEM.

Compared to CTRL, AB had no effect on EET or an effect on cardiac, metabolic, perceptual, ventilatory, breathing pattern, and/or operating lung volume parameters at rest or during exercise (Table 2, Figures 1, 2).

**Table 2:** **Effect of abdominal binding (AB) on physiological and perceptual responses to constant-load cycle endurance exercise testing at 85% of symptom-limited peak incremental power output (equivalent to 227 ± 17 W) in healthy men**.

**Parameter**	**REST**		**ISO-TIME**		**PEAK**	
	**Control**	**AB**	**Control**	**AB**	**Control**	**AB**
Exercise time, min	0 ± 0	0 ± 0	7.5 ± 0.8	7.5 ± 0.8	9.9 ± 1.0	8.9 ± 1.1
Breathlessness, Borg 0–10 units	0.2 ± 0.2	0.3 ± 0.2	6.1 ± 0.6	6.8 ± 0.6	8.1 ± 0.7	8.3 ± 0.7
Leg Discomfort, Borg 0–10 units	0.0 ± 0.0	0.0 ± 0.0	6.9 ± 0.7	7.3 ± 0.6	8.1 ± 0.7	8.3 ± 0.5
$\dot{\text{V}}$O_2_, ml × kg × min^−1^	5.6 ± 0.2	5.1 ± 0.5	51.6 ± 2.3	51.5 ± 2.5	53.6 ± 2.1	52.4 ± 2.5
$\dot{\text{V}}$CO_2_, ml × kg × min^−1^	4.4 ± 0.2	4.7 ± 1.0	53.3 ± 1.9	53.8 ± 2.2	53.9 ± 1.7	53.9 ± 2.1
CO, L × min^−1^	5.9 ± 0.3	5.5 ± 0.4	20.3 ± 1.0	20.6 ± 1.0	21.3 ± 1.1	21.3 ± 1.0
HR, beats × min^−1^	78.4 ± 3.2	76.3 ± 3.2	172.7 ± 3.4	174.1 ± 3.3	182.6 ± 2.3	178.7 ± 2.9
SV, ml	75.6 ± 4.3	70.9 ± 3.8	117.9 ± 6.4	118.5 ± 6.1	116.6 ± 6.5	119.8 ± 6.0
$\dot{\text{V}}$_*E*_, L × min^−1^	12.5 ± 0.9	11.2 ± 0.9	116.4 ± 7.3	120.7 ± 7.7	133.4 ± 8.0	130.6 ± 8.6
V_*T*_, L	0.90 ± 0.09	0.74 ± 0.06	2.96 ± 0.17	2.90 ± 0.21	2.72 ± 0.17	2.73 ± 0.19
*f*_*R*_, breaths × min^−1^	15.2 ± 1.1	15.7 ± 0.8	39.9 ± 2.0	42.8 ± 2.4	50.4 ± 3.6	49.8 ± 4.0
IC, L	3.38 ± 0.17	3.62 ± 0.16	3.84 ± 0.22	3.82 ± 0.20	3.68 ± 0.19	3.81 ± 0.23
IRV, L	2.48 ± 0.19	2.87 ± 0.15	0.89 ± 0.14	0.92 ± 0.18	0.96 ± 0.14	1.08 ± 0.17
EMGdi,rms, μV	22.5 ± 1.9	27.4 ± 3.7	129.2 ± 13.3	120.0 ± 11.8	150.7 ± 28.1	123.2 ± 14.7
EMGdi%max	10.4 ± 1.1	13.1 ± 2.1	56.0 ± 2.8	53.0 ± 2.9	61.9 ± 5.2	53.3 ± 3.4
End-expiratory Pes, cmH_2_O	−7.3 ± 0.7	−5.0 ± 0.7	−5.4 ± 1.1	−4.3 ± 1.2	−6.0 ± 1.1	−5.3 ± 0.9
Pes,tidal, cmH_2_O	6.1 ± 0.9	4.8 ± 0.6	31.6 ± 2.9	31.2 ± 2.6	35.0 ± 3.0	34.5 ± 2.8
Peak inspiratory Pes, cmH_2_O	−11.7 ± 1.8	−8.4 ± 0.8	−23.6 ± 1.9	−21.5 ± 1.7	−24.3 ± 1.9	−22.0 ± 1.9
Peak expiratory Pes, cmH_2_O	−5.6 ± 0.7	−3.5 ± 0.8	8.1 ± 2.3	9.8 ± 1.5	10.8 ± 2.2	12.5 ± 1.7
End-expiratory Pga, cmH_2_O	7.7 ± 1.2	12.3 ± 1.2^*^	14.3 ± 1.4	17.8 ± 0.9	14.4 ± 1.3	19.1 ± 1.1^*^
Pga,tidal, cmH_2_O	5.2 ± 0.5	9.0 ± 0.8	17.3 ± 1.7	18.0 ± 1.3	19.2 ± 1.6	17.1 ± 1.1
Peak inspiratory Pga, cmH_2_O	6.5 ± 1.3	11.7 ± 1.2^†^	4.3 ± 1.2	14.0 ± 1.0^†^	4.3 ± 1.1	14.8 ± 0.9^†^
Peak expiratory Pga, cmH_2_O	11.7 ± 1.6	20.7 ± 1.5^*^	21.6 ± 1.9	32.0 ± 1.7^†^	23.5 ± 1.9	31.9 ± 1.6^*^
End-expiratory Pdi, cmH_2_O	15.1 ± 0.9	17.3 ± 0.9	19.6 ± 1.3	22.0 ± 1.2	20.4 ± 1.2	24.5 ± 1.3
Pdi,tidal, cmH_2_O	9.6 ± 0.9	12.8 ± 1.0	20.4 ± 1.5	36.9 ± 2.3^†^	21.4 ± 1.5	35.8 ± 2.1^†^
Peak inspiratory Pdi, cmH_2_O	22.9 ± 1.2	28.8 ± 1.6	32.0 ± 1.8	50.2 ± 2.6^†^	32.4 ± 1.6	49.5 ± 2.5^†^
Peak expiratory Pdi, cmH_2_O	13.4 ± 1.2	16.0 ± 1.1	11.5 ± 1.1	13.4 ± 1.1	11.0 ± 1.2	13.7 ± 1.1
Pdi,tidal:EMGdi,rms, cmH_2_O × μV^−1^	0.44 ± 0.04	0.54 ± 0.06	0.17 ± 0.01	0.33 ± 0.03^†^	0.17 ± 0.01	0.33 ± 0.03^†^

*Values are means ± SEM. $\dot{\text{V}}$O_2_ and $\dot{\text{V}}$CO_2_, rate of oxygen consumption and carbon dioxide output, respectively; CO, cardiac output; HR, heart rate; SV, stroke volume; $\dot{\text{V}}$_E_, minute ventilation, V_T_, tidal volume; f_R_, respiratory frequency; IC, inspiratory capacity; IRV, inspiratory reserve volume; EMGdi,rms, root mean square of the crural diaphragm electromyogram; EMGdi%max, root mean square of the crural diaphragm electromyogram expressed as a percentage of the maximal voluntary room mean square of the crural diaphragm electromyogram; Pes, Pga and Pdi, esophageal, gastric, and transdiaphragmatic pressure, respectively; Pes,tidal, tidal esophageal pressure swing; Pga,tidal, tidal gastric pressure swing; Pdi,tidal, tidal transdiaphragmatic pressure swing; Pdi,tidal:EMGdi,rms, tidal transdiaphragmatic swing-to-root mean square of the diaphragmatic electromyogram ratio—an index of neuromuscular efficiency of the diaphragm*.

* p < 0.05 and

† *p ≤ 0.01 vs. Control*.

The relative contributions of breathlessness (AB, 46 ± 8% vs. CTRL, 40 ± 7%; *p* = 0.592) and leg discomfort (AB, 54 ± 8% vs. CTRL, 60 ± 7%; *p* = 0.592) to exercise cessation were not different under AB vs. CTRL conditions. The distribution of reasons for stopping exercise were also similar between-tests: Breathlessness: AB, *n* = 1 vs. CTRL, *n* = 1; Leg discomfort: AB, *n* = 0 vs. CTRL, *n* = 1; Combination of breathlessness and leg discomfort: AB, *n* = 10 vs. CTRL, *n* = 9. The majority of participants self-selected phrases alluding to a heightened sense of “*work/effort of breathing*” to describe their breathlessness at end-exercise under both AB and CTRL conditions; for example, “*My breathing is heavy*” (AB, 100% vs. CTRL, 92%) and “*My breathing requires more work*” (AB, 92% vs. CTRL, 100%).

### Diaphragmatic EMG and respiratory pressures

Maximal voluntary EMGdi,rms was not significantly different under AB vs. CTRL conditions: 227 ± 19 vs. 234 ± 25 μV, respectively (*p* = 0.727). Peak inspiratory Pes values recorded during serial IC maneuvers did not change significantly from rest (AB, −34.2 ± 3.4 cmH_2_O; CTRL, −34.5 ± 2.2 cmH_2_O) and throughout exercise (e.g., AB at end-exercise, −34.8 ± 2.9 cmH_2_O; CTRL at end-exercise, −36.1 ± 2.6 cmH_2_O) both within and between conditions. Peak inspiratory Pdi values recorded during serial IC maneuvers (Pdi,IC) performed at rest and throughout exercise were significantly increased by 18.5–22.2 cmH_2_O (or 43–53%) under AB vs. CTRL conditions; for example, AB, 70.3 ± 6.0 cmH_2_O vs. CTRL, 48.5 ± 4.9 cmH_2_O at rest (*p* < 0.001); and AB, 59.2 ± 4.0 cmH_2_O vs. CTRL, 40.2 ± 2.8 cmH_2_O at end-exercise (*p* < 0.001).

**Figure 3:**
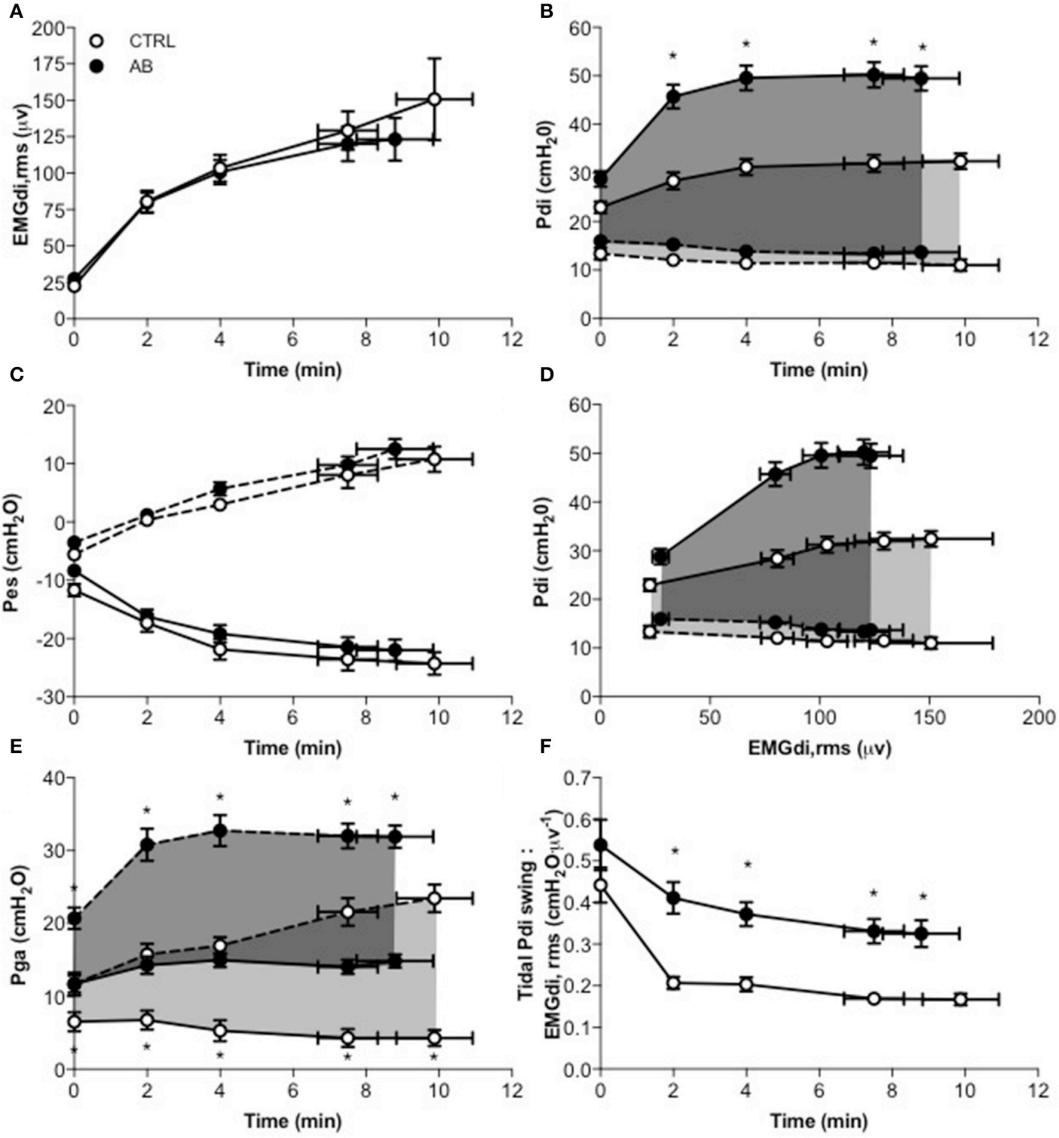
**Effect of abdominal binding (AB) vs. control (CTRL) on (A)** root mean square of the crural diaphragm electromyogram (EMGdi,rms), **(B)** transdiaphragmatic pressure (Pdi), **(C)** esophageal pressure (Pes), **(D)** Pdi vs. EMGdi,rms, **(E)** gastric pressure (Pga), and **(F)** tidal Pdi swing-to-EMGdi,rms ratio responses during constant-load cycle endurance exercise testing at 85% of peak incremental power output in healthy men. Values are means ± SEM. ^*^p < 0.05 vs. CTRL. Dashed lines denote expiratory Pdi, Pes and Pga.

Compared with CTRL, AB had no effect on either EMGdi,rms (Figure 3A) or Pes (Figure 3C) responses during exercise (Table 2). As expected, peak tidal inspiratory Pga (Pga,inspir) and peak tidal expiratory Pga (Pga,expir) were consistency higher at rest and during exercise with vs. without AB (Table 2, Figure 3E). Compared with CTRL, AB increased Pdi,tidal and peak tidal inspiratory Pdi (Pdi,inspir) at rest and during exercise; for example, by +16.5 cmH_2_O at rest and by +28.2 cmH_2_O during exercise at isotime with vs. without AB (Table 2, Figure 3B). Furthermore, AB was associated with a marked increase in the magnitude of the exercise-induced rise in Pdi,tidal and Pdi,inspir (Table 2, Figure 3B): the respective increases in Pdi,tidal and Pdi,inspir from rest to isotime during exercise were ~315 and 223% greater with vs. without AB. As illustrated in Figure 3D, Pdi,tidal and Pdi,inspir were much higher at any given EMGdi,rms during exercise with vs. without AB, indicating enhanced neuromuscular efficiency of the diaphragm. Indeed, AB increased the Pdi,tidal:EMGdi,rms ratio by an average of 85–95% at each measurement time during exercise (Table 2, Figure 3F).

## Discussion

The main finding of this study was that AB sufficient to increase intra-abdominal pressure by an average of 6.6 cmH_2_O at rest enhanced neuromuscular efficiency of the diaphragm during exercise, but had no effect on exercise endurance nor an effect on cardiac, metabolic, ventilatory, breathing pattern, dynamic operating lung volume, and perceptual responses to exercise in healthy young men.

In keeping with the results of earlier AB studies in health (Hussain et al., 1985) and SCI (Hart et al., 2005; West et al., 2012a, 2014a,b), the increased Pdi,tidal, Pdi,inspir, and Pdi,IC responses observed at rest and during exercise with vs. without AB were mechanistically linked to increased intra-abdominal pressures (i.e., Pga,ee and Pga,expir). The increased intra-abdominal pressures associated with AB effectively shift the abdominal contents toward the diaphragm (cephalad), thereby increasing both insertional and appositional forces of the diaphragm on the lower rib cage (Wilson and De Troyer, 2013; Koo et al., 2015). By shifting the diaphragm cephalad, AB also lengthens diaphragm muscle fibers and optimizes its length-tension relationship (Koo et al., 2015). As a result, the diaphragm initiates its inspiratory contraction at a longer length, thus generating a greater pressure at any given level of muscle activation, reflecting enhanced diaphragmatic contractility (De Troyer, 1983). Abdominal binding may further enhance pressure-generating capacity of the diaphragm by improving (reducing) abdominal compliance, thus impeding diaphragmatic descent at the costal fibers during inspiration and minimizing muscle fiber shortening, i.e., maintaining the muscle length on a more favorable region of the length-tension curve (De Troyer, 1983; Hart et al., 2005; Koo et al., 2015). Finally, by increasing intra-abdominal pressures and decreasing abdominal compliance, AB may increase the inflationary action of the diaphragm on the lower rib cage by increasing the zone of apposition and improving the diaphragm's ability to lift and expand the lower rib cage (De Troyer, 1983; Koo et al., 2015). The combination of these mechanically advantageous changes to the shape and configuration of the diaphragm are most likely responsible for the 85–95% increase in neuromuscular efficiency of the diaphragm observed during exercise with vs. without AB.

Although AB increased diaphragmatic contractility/pressure-generating capacity, it had no demonstrable effect on EMGdi,rms, Pes, $\dot{\text{V}}$_E_, breathing pattern, and dynamic operating lung volume responses to exercise. These findings are similar to those of earlier AB studies by Hussain et al. (1985) in health and by West et al. (2014b) in SCI, and presumably reflect the fact that AB had no untoward effect on expiratory flow generation during exercise (as evidenced by relative preservation of the relationship between exercise-induced increases in peak tidal expiratory Pes and peak expiratory flow) or an effect on exercise-induced increases the rate of CO_2_ production, which is the proximate source of increased ventilatory requirements during exercise. It could be argued that the increased intra-abdominal pressures associated with AB may have hindered descent of the diaphragm into the abdomen at rest and particularly during exercise when ventilatory requirements were ~13-fold higher than at rest. If this was true, then maximal voluntary EMGdi,rms as well as the magnitude of exercise-induced increases in EMGdi,rms should have been consistently higher under AB vs. CTRL conditions. However, this is not what we observed in our study nor what Hussain et al. (1985) reported in their AB study of five healthy men.

In the setting of a relatively preserved EMGdi,rms, $\dot{\text{V}}$_E_, breathing pattern and dynamic operating lung volume response to exercise with vs. without AB, we speculate that the disparate effect of AB on Pdi and Pes responses to exercise reflected “off-loading” of the inspiratory action(s) of the rib cage muscles. In other words, by increasing Pdi,inspir and thus Pdi,tidal responses to exercise, AB effectively decreased the rib cage muscles' relative contribution to any given level of negative intrathoracic pressure development throughout inspiration during exercise. Additional research with simultaneous measures of accessory inspiratory muscle EMG activity is needed to confirm this postulate.

In the absence of changes in EMGdi,rms, $\dot{\text{V}}$_E_, breathing pattern, expiratory flow generation, and dynamic operating lung volume responses to exercise, isolated and acute improvements in neuromuscular efficiency of the diaphragm during exercise with vs. without AB had no effect on exercise endurance and/or exertional breathlessness. These findings support the view that, in healthy young adults: (1) respiratory mechanical/muscular factors do not likely contribute to the limits of exercise tolerance; and (2) progressive neuromuscular uncoupling of the diaphragm is not likely a proximate source of exertional breathlessness. Nevertheless, the results of our study provide a physiological rationale for future examination of AB as a potentially effective non-pharmacological means of improving exercise tolerance in pathophysiological disease states where neuromuscular uncoupling of the diaphragm has been mechanistically linked to a heightened perception of exertional breathlessness, most notably in patients with COPD (Laghi et al., 1998). Interestingly, a case report by Celli et al. (1985) found that AB sufficient to increase Pga,ee from 4 to 12 cmH_2_O was associated with objective and potentially clinically meaningful improvements in diaphragmatic function, exercise tolerance, and breathlessness in a symptomatic patient with severe COPD and a large midline hernia of the anterior abdominal wall.

The collective results of studies by Vivier et al. (2006), Aliverti et al. (2009, 2010), and Uva et al. (2015) suggest that AB, by increasing intra-abdominal pressure and/or the abdominal circulatory pump action of the diaphragm and abdominal muscles, has the potential to improve cardiac function at rest and during exercise by increasing central venous return from the splanchnic venous circulation. In our study, however, AB had no demonstrable effect on impedance cardiograph-derived estimates of CO and SV at rest and during exercise, which is in agreement with West et al. (2012a) who reported no effect of AB on echocardiography-derived measures of cardiac function at rest (e.g., CO, SV, end-diastolic volume, end-systolic volume, ejection fraction) in eight healthy adults. We speculate that AB-induced increases in intra-abdominal pressure and/or the abdominal circulatory pump action of the diaphragm and abdominal muscles were of insufficient magnitude(s) to shift large enough quantities of blood from the splanchnic to central venous circulation to enhance cardiac function at rest and during exercise in our participants.

In summary, the increased intra-abdominal pressures associated with AB enhanced neuromuscular efficiency of the diaphragm by 85–95% during high-intensity constant-load cycle endurance exercise testing in healthy men. Additional research is recommended to examine potential benefits of AB on exertional symptoms in clinical populations where diaphragmatic weakness/dysfunction has been implicated as a source of physical activity-related breathlessness and exercise intolerance.

## Author contributions

SA, DC, RG, and DJ contributed to the conception of the study as well as to data collection, analysis, and interpretation. CM contributed to data collection and analysis. SA and DJ wrote the manuscript, with critical input from all other authors. All authors read and approved the final version of the manuscript.

### Conflict of interest statement

The authors declare that the research was conducted in the absence of any commercial or financial relationships that could be construed as a potential conflict of interest.
